# Assessment of Proximal Protection Usage by Dental Students During Class II Cavity Preparations: An In Vivo Pilot Study

**DOI:** 10.7759/cureus.47550

**Published:** 2023-10-23

**Authors:** Rozana A Al-Bukhary, Alaa I Mannaa

**Affiliations:** 1 Department of Restorative Dentistry, King Abdulaziz University, Jeddah, SAU

**Keywords:** clinical supervision, medical education, cavity preparation, proximal protection, restorative dentistry

## Abstract

Background: Class II cavity preparations in restorative dentistry pose a risk of iatrogenic damage to adjacent teeth as they could increase their susceptibility to caries and the need for additional restorative procedures. While previous research has explored this issue through in vitro and limited in vivo investigations, the direct clinical observation of proximal protection usage to prevent iatrogenic damage during class II cavity preparations is still limited.

Aim: This in vivo study aimed to assess the prevalence of proximal protection usage and extent of iatrogenic damage induced to adjacent surfaces upon occurrence during class II cavity preparations via direct visual inspection under magnification.

Materials and methods: Data were collected from restorative dentistry consultants supervising fourth-year undergraduate students. Information regarding cavity preparations, proximal protection usage, and iatrogenic damage was gathered through an electronic evaluation form via direct clinical observations once class II cavity preparations were finished. Statistical analyses, including ordinal logistic regression models, were employed to investigate associations and compute odds ratios (ORs).

Results: We examined 82 teeth adjacent to class II cavity preparations in in vivo settings. The prevalence of proximal protection use was 72%, while the prevalence of iatrogenic damage was 17.1% overall, increasing to 47.8% when not using protection and 5.1% when using protective measures. Polishing and restoration of the induced damage were the primary management approaches. The use of protective measures, particularly matrix bands, significantly reduced the risk of iatrogenic damage (P < 0.05). Several factors, including arch, tooth surface, time, operator gender, and the state of the adjacent tooth before treatment, were examined but did not yield statistically significant associations.

Conclusion: Our study shows that most undergraduate dental students use proximal protection during class II cavity preparations, which are significant in reducing iatrogenic damage to the adjacent tooth. However, the literature shows that general practitioners often do not use proximal protection. The use of proximal protection should be reinforced and even required for the successful and safe treatment of proximal cavities. Future research with larger and more diverse samples is needed to understand the barriers to the use of proximal protection and develop interventions to promote its adoption.

## Introduction

Class II cavity preparations for proximal caries may be frequently accompanied by iatrogenic damage to adjacent teeth [1,2]. Consequently, this can increase the risk of caries initiation or progression in the proximal surface in contact with the class II restoration and could ultimately warrant restorative treatment for the adjacent tooth [3,4]. The possibility of iatrogenic damage to proximal surfaces was initially reported by Boyde and Knight [5]. Afterward, a few “in vitro” studies showed that the frequency of this type of damage was high [4,6-9]. Such damage can have one of several morphological presentations, including vertical grooves in the long axis of the tooth (most common), abrasions, horizontal scorelines, scratches or grooves, local indentations, or a combination of these [1-4,8-10]. Lussi and Gygax divided iatrogenic proximal damage into superficial and severe defects exposing deeper layers of dental tissues [3]. Moopnar and Faulkner reported that proximal iatrogenic damage occurred significantly lower in the anterior than posterior teeth [9]. In addition, they found that distal surfaces were more prone to damage than mesial ones. However, they found no differences between the frequency of damage to the maxillary and mandibular teeth. In majority of such studies, damage assessment was evaluated in either typodont teeth or impressions of cavities prepared on natural teeth under 3x magnification or scanning electron microscopy. Only one “in vivo” study has examined such iatrogenic damages on natural teeth by taking impressions immediately after occurrence for evaluation under scanning electron microscopy [2]. Collectively, the results of available “in vitro” and “in vivo” studies indicate that 64% to 97% of enamel surfaces of teeth adjacent to class II cavity preparations might be damaged. Research has shown that such proximal iatrogenic injuries in the enamel can predispose the affected surfaces to the number of sequelae, including increased permeability to acidic attacks and plaque retention predisposing to caries development or progression [4,9,11-15]. Moreover, such a damaged proximal enamel may appear as interproximal radiolucency, which could ultimately be misdiagnosed as carious cavitations, leading to unwarranted restorative treatment [1,14].

Proximal protection strategies have ranged over the years from the sole use of tooth separators (wedges) or metal matrix bands (t-bands and Tofflemire bands or preshaped and contoured sectional matrices) to their combined use and have further developed into the use of fender wedges. Earlier studies showed that despite the suitability of metal matrix bands, they were rarely used to prevent iatrogenic damage during class II cavity preparations [1,3,5]. The same stood for traditional tooth separators [1,2]. There was also a direction toward modifying proximal cavity preparation techniques to minimize proximal surface damage. One study quantified proximal tooth damage using three different preparation techniques: diamond bur alone, diamond bur combined with the EVA system and the Cavishape file, and diamond bur combined with an axial margin trimmer [3]. However, no significant differences were observed between the methods under study. Meanwhile, another study suggested minimizing proximal surface damage by preparing proximal cavities using less invasive modalities, such as the atraumatic restorative treatment, which is a minimally invasive technique for restoring teeth by means of hand instrumentation for decay removal and placement of fluoride-releasing adhesive materials, such as glass ionomer, or utilization of caries detection dyes as opposed to burs [16]. A later study then showed that the use of proximal guards, such as stainless-steel matrix bands and protective wedges, reduced iatrogenic damage to proximal surfaces during preparation with high-speed rotary instruments [17]. Nevertheless, nothing in the literature mandates the use of proximal guards to preserve neighboring teeth during restorative procedures, rendering their use optional or advisable. Moreover, this topic is certainly addressed in undergraduate dental curricula worldwide, but there are several concerns that surfaces, such as the reinforcement of its use by supervisors during clinical sessions, the efficacy of its monitoring during such sessions, the use of clinical performance and operative procedural experience rubrics that incorporate it as an item for evaluation, and its inclusion as a criterion for success or failure in an operative/restorative clinical competency examination.

To our knowledge, no studies have determined the prevalence of proximal protection to prevent iatrogenic proximal damage via direct visual clinical observations upon occurrence of the damage. Thus, the aim of this “in vivo” study was to investigate the prevalence of proximal tooth protection use during class II cavity preparations in fourth-year undergraduate dental students and the extent of induced iatrogenic damage to the proximal surfaces adjacent to such preparations.

## Materials and methods

Study design

Ethical approval for the study was obtained from the Faculty of Dentistry at King Abdulaziz University (KAUFD), Jeddah, Saudi Arabia (approval no. 4292996, date: 10/03/2022). Consultants from the Department of Restorative Dentistry at KAUFD assigned to supervise fourth-year undergraduate students (the first year of clinical training in restorative dentistry) were invited to fill in an electronic evaluation form designed to gather information regarding class II cavity preparations done by the fourth-year students during their clinical sessions. This study did not alter the treatment plan for any patient, nor did it result in the collection of any identifying information; as such, and according to the Federal Policy for the Protection of Human Subjects (Common Rule) and according to the institutional review board’s guidelines, no consent was required for this cross-sectional study.

The form included the following information: type of treated tooth (maxillary or mandibular, premolar or molar); tooth surface being prepared (mesial, distal, occluso-mesial, occluso-distal, and mesio-occluso-distal); condition of adjacent proximal surface (sound, incipiently carious, cavitated, optimally restored, and defectively restored); the order of the student’s class II cavity preparation defined as 1st, 2nd, 3rd, 4th, 5th, or 6th attempt; type of proximal protection used (none used, only wedge, only matrix band, matrix band with wedge, and other means, such as fender wedge); induced surface damage to the adjacent tooth (no surface damage detected visually and/or by means of a dental probe, surface damage detected visually and/or by means of a dental probe either in the form of enamel scratching, shell, or slicing, depth of proximal damage measured by periodontal probe (no surface damage, surface roughness, cavitation less than 0.5 mm, cavitation more than 0.5 mm); and management of surface damage (no management needed, polishing, restoration placement, or restoration replacement).

The management of the damage was dependent on the type of damage detected visually and/or using the dental probe. If the damage indicated the need for the restoration of a newly formed cavity, the appropriate restorative measures were taken. Meanwhile, when the damage compromised the integrity of an existing dental restoration, the management involved either repairing it, if possible, or totally replacing it. The International Caries Detection and Assessment System (ICDAS) was used to clinically score the carious proximal surface adjacent to the class II cavity. KAUFD uses a modified version of the ICDAS for evaluating caries severity, where instead of having seven codes ranging from 0 to 6, it uses only five (0 sound, 1 enamel lesion visible only after drying, 2 enamel lesion visible without drying, 3 shadowing of dentin with or without cavitation, 4 extensive lesion with visible dentin). In addition, as a standard of care at KAUFD, a preoperative bitewing is mandatory prior to class II cavity preparations for the detection of incipient proximal enamel lesions and was used to aid in the evaluation of the original condition of the proximal surface adjacent to the class II cavity preparation. All clinical instructors were asked to use 2.5x magnification loupes during the evaluation process.

Statistical analysis

Ordinal logistic regression models were employed to investigate associations and compute odds ratios (OR) concerning the impact of using protective measures and the attempt number (student's experience) on the likelihood of causing iatrogenic damage to the neighboring tooth. Model fitting was maintained at P < 0.05, while the goodness-of-fit was maintained at P > 0.05. The dependent variable, iatrogenic damage, was treated as an ordinal variable encompassing multiple categories, ranging from "no damage" to "cavitation exceeding 0.5 mm." The use of protection was evaluated both as a binary variable (used/did not use) and based on the specific type of protection employed. Additional independent variables, such as the time of the procedure, the gender of the operator, and the state of the adjacent tooth prior to the procedure, were assessed using different models. P-values < 0.05 were considered statistically significant. All analyses were conducted using IBM SPSS Statistics for Windows, version 28 (released 2021; IBM Corp., Armonk, New York, United States).

## Results

In this cross-sectional study, we examined a total of 82 teeth adjacent to class II cavity preparations to assess the presence of iatrogenic damage. Table 1 summarizes all descriptive statistics. The distribution of both the gender of the students and the time of the procedure was approximately equal. The prevalence of proximal protection use was 72%, mostly in the form of a matrix band (50%). The prevalence of iatrogenic cavitation in the adjacent tooth was found to be 17.1% when surface roughness was included. The primary management approaches for these complications were polishing alone or restoration of the traumatized adjacent tooth. The majority of treated teeth were located in the maxillary arch (33% in quadrant 1 and 31% in quadrant 2). The predominantly treated teeth were premolars (77%), with the second premolar being the most frequently treated tooth (51%). Most iatrogenic damage occurred during treatment of the mesial surface (73%), primarily in the teeth located in quadrant 2 (46%).

**Table 1:** Descriptive statistics of the students and the restored teeth *: a combination of matrix band and wooden wedge or other means, such as fender wedge. MOD: mesio-occluso-distal, ICDAS: International Caries Detection and Assessment System

Variable		Frequency	Percentage
		n	(%)
Time of procedure	Morning	40	48.8
	Afternoon	42	51.2
Operator gender	Male	40	48.8
	Female	42	51.2
Surfaces included in cavity preparation	Mesial	26	31.7
	Distal	38	46.3
	Occluso-mesial	6	7.3
	Occluso-distal	10	12.2
	MOD	2	2.4
Original condition of the adjacent tooth	Sound	44	53.7
	ICDAS score 1 or 2	20	24.4
	ICDAS score 3 or 4	12	14.6
	Restored: non-defective	3	3.7
	Restored: defective	3	3.7
Proximal protection	None	23	28
	Only wedge	8	9.8
	Only matrix band	41	50
	Combination or other means*	10	12.2
Type of induced damage	None	67	81.7
	Present (enamel scratching, shell, or slicing)	15	18.3
Depth of induced damage measured with a probe	No surface damage	68	82.9
	Surface roughness without cavitation	3	3.7
	Cavitation < 0.5 mm	6	7.3
	Cavitation > 0.5 mm	5	6.1
Management of induced damage	None needed	70	85.4
	Only polishing	6	7.3
	Restoration placement or repair	6	7.3

Ordinal logistic regression models revealed that the students who used protective measures during their class II cavity preparations were significantly less likely to cause iatrogenic damage to the neighboring tooth (β = -2.78; OR = 0.06; Wald χ2 = 14.27; 95% confidence interval (CI), -4.221 to -1.337; P < .05). Further analysis indicated that using matrix bands as a protective measure during cavity preparations was associated with a significantly reduced risk of iatrogenic proximal damage (β = -2.39; OR = 0.09; Wald χ2 = 10.43; 95% CI, -3.85 to -0.94; P < .05) compared to using no protection, wedges for protection, or a combination of wedges and matrix bands (Table 2). Other factors, including the time of the procedure, the gender of the operator, and the state of the adjacent tooth before treatment, were examined using regression models, but none yielded statistically significant associations with the risk of iatrogenic proximal damage (P > 0.05) (Table 3).

**Table 2:** Regression model for the number of attempts and the use of protection †: reference, *: P-value is significant at < 0.05.

Parameter			Estimate	Odds ratio	95% confidence Interval	
					Lower bound	Upper bound
Attempt number			0.213	1.24	-0.234	0.659
Protection use	Used		-2.779*	0.06	-4.221	-1.337
	Did not use^†^					
Protection type	None^†^					
	Wedge		-20.682	0.00	-20.682	-20.682
	Matrix band		-2.394*	0.09	-3.847	-0.941
	Combination		-20.647	0.00	-20.647	-20.647

**Table 3:** Regression model for the day and time of the procedure, gender of the operator, and tooth state before †: reference. ICDAS: International Caries Detection and Assessment System

Parameter		Estimate	Odds ratio	95% confidence interval	
				Lower bound	Upper bound
Time	Morning	-0.235	0.79	-1.388	0.918
	Afternoon^†^				
Gender	Female	-0.662	0.52	-1.843	0.52
	Male^†^				
Original condition of the adjacent tooth	Sound^†^				
	ICDAS score 1 or 2	-0.865	0.42	-2.5	0.769
	ICDAS score 3 or 4	-0.353	0.70	-2.07	1.364
	Restored - non defective	-18.526	0.00	-18.526	-18.526
	Restored - defective	0.985	2.68	-1.344	3.314

## Discussion

In this in vivo study, we aimed to investigate the prevalence of proximal tooth protection use during class II cavity preparations in fourth-year undergraduate dental students and the extent of induced iatrogenic damage to the proximal surfaces adjacent to such preparations. Our findings revealed a high prevalence of proximal protection use (72%). Moreover, they revealed the prevalence of iatrogenic proximal damage, as observed clinically, to be 17.1%. Notably, when protective measures were employed, this prevalence dropped significantly to 5.1%, contrasting with a prevalence of 47.8% in cases where dental students performed cavity preparations without protection. This difference underscores the crucial role of proximal protection in reducing iatrogenic damage during class II preparations. It is essential to acknowledge that our study participants consisted of dental students in their first clinical dentistry year (4th undergraduate year), a stage of training during which students tend to exhibit heightened caution during clinical procedures. This heightened caution likely explains the increased use of proximal protection among these students. Moreover, clinical supervisors tend to verify the use of proximal protection by students before, during, and after the procedure. However, it is important to note that this trend may change as these individuals progress in their careers and become less supervised. This shift in behavior is consistent with findings from more experienced practitioners, who have been documented to encounter nearly a threefold increase in iatrogenic damage to proximal tooth surfaces adjacent to class II cavities compared to undergraduate students [1]. Therefore, our study highlights the importance of continuous education and reinforcement of best practices, such as the consistent use of proximal protection, throughout a dental practitioner's career.

Comparatively, our observed prevalence of iatrogenic damage (47.8%) in the absence of protection falls below previously reported figures, ranging from 60% to 100% in in vivo studies [1,3,4] and 74% in in vitro studies [17]. These earlier studies often involved more experienced practitioners, and the discrepancy in results further emphasizes the impact of experience and training on the use of protective measures.

This can be especially evident when looking at the outcomes of the study by Milic et al. who found the prevalence of iatrogenic damage to be 50% and 46% when using either matrix bands or wedges as protection, respectively, as their study is not only in vitro and uses a small sample size, but also half of the operators were experienced dental practitioners [17]. Comparatively, both percentages reported by Milic when using protection are significantly higher than our reported prevalence (5.1%), which falls to 0% with the use of either wedges combined with bands or wedges only. Bands usually cover the full height of the proximal surface, which may give a false feeling of security. By contrast, wedges are usually positioned inferior to the preparation, which may warrant augmented feeling of cautiousness. This, combined with the triangular and tapering design of wedges, which leads to an increase in the interproximal space, might have led to the 0% prevalence reported in our study. Noteworthy is the extended benefits of using wedges in class II restorations to not only mitigate iatrogenic damage but to also guarantee the correct restoration of the proximal surface by restoring natural proximal contour and avoiding overhang restorations [18,19]. Furthermore, our logistic regression analysis indicates that the use of protective measures, particularly matrix bands alone, significantly reduces the risk of iatrogenic damage when compared to their combined use with wedges or the use of wedges alone. Nevertheless, it is important to acknowledge the limitations of our study, particularly the small sample sizes for groups utilizing protective measures other than matrix bands, which limits the statistical power. In addition, the rarity of iatrogenic damage in the presence of protection makes statistical interpretation challenging and necessitates further investigation to determine its significance.

Regarding other factors, our data did not identify significant effects associated with an arch or tooth surface. However, we observed that the second quadrant exhibited the highest incidence of iatrogenic damage, accounting for 35.6% of all incidents. This observation aligns with findings from previous studies, which reported a higher incidence of iatrogenic damage in maxillary teeth compared to mandibular arches [1]. The unique challenges posed by maxillary posterior teeth preparation, often performed indirectly using oral mirrors, may explain this trend. Distinctly, observing the distal surface of the adjacent tooth while working on a mesial cavity requires certain finesse and experience compared to observing the mesial surface while working on a distal cavity, which explains the higher percentage of incidents occurring while treating the mesial surface (57.1%) compared to the distal or mesio-occlusal-distal cavities. Our study also noted a reduced incidence during morning sessions and among female operators, although these observations lacked statistical significance. Furthermore, the state of the adjacent surface prior to the procedure did not seem to affect the rate of iatrogenic damage. Our initial hypothesis posited that operators, when made aware of the pre-existing damage or restoration on an adjacent surface, might exhibit either heightened or lowered caution during the procedure. However, the assessment of this factor would benefit from future investigations with larger sample sizes to yield more conclusive insights.

## Conclusions

We found the use of proximal protection to be prevalent among undergraduate students. Consequently, we found iatrogenic damage to occur significantly less when proximal protection was used. In our dental institute, clinical supervisors ensure the use of proximal protection at all stages of treatment. However, this supervision is lost once students graduate, and prior research has shown proximal protection use to be far less among practitioners, with a far higher incidence of iatrogenic damage. As such, our study highlights not only the clinical significance of proximal protection use but also the importance of enforcing such standards of care. We believe that using proximal protection should be a mandatory step and that students should be evaluated on it, as the occurrence of iatrogenic damage inherently lowers the standard of care that our patients are receiving. Future research with larger and more diverse samples is needed to understand the barriers to the use of proximal protection and develop interventions to promote its adoption.
